# Standardising the preparation for, and delivery of care during, and after critical incidents: a Faculty of Sport and Exercise Medicine UK publication

**DOI:** 10.3389/fspor.2026.1864572

**Published:** 2026-05-21

**Authors:** Neil Heron, Steffan Griffin

**Affiliations:** 1 Centre for Public Health, Queen’s University Belfast, Belfast, United Kingdom; 2 SailGP, London, United Kingdom; 3 Physical Activity for Health Research Centre, University of Edinburgh, Edinburgh, United Kingdom

**Keywords:** athlete safety and welfare, critical incidents, emergency action planning, post-traumatic care, sports medicine

## Abstract

Participation in sport provides substantial physical and mental health benefits; however, it also carries a risk of rare but high-impact critical incidents. This policy brief outlines a practical, standardised framework for the preparation, management, and follow-up of such incidents in sport. Drawing on current evidence and expert consensus, we emphasise the importance of robust, rehearsed emergency action plans tailored to specific environments, alongside structured post-incident care incorporating communication, debriefing, and psychological support through the “5Cs” approach. This publication is supported by the Faculty of Sport and Exercise Medicine UK (FSEM), reflecting its alignment with current best practice in sport and exercise medicine. By integrating prevention, acute response, and rehabilitation, this work provides actionable guidance to improve athlete safety and promote consistent, high-quality care across diverse sporting contexts.

## Introduction

The benefits of physical activity, which sport provides, are well known (1). Being physically active reduces the risk of several non-communicable diseases, including cardiovascular disease, cancer, and non-insulin-dependent diabetes mellitus (2–4). Sport is also known to provide a range of health and wellbeing benefits, including improved mental health and wellbeing (5, 6).

Although the benefits of being physically active through sport are well understood, participating in sport also carries its own risks. Each sport has its own associated injury burden, and the risk of injury generally correlates with the level of athletic participation (7, 8). Similarly, the risk of sudden cardiac arrest (SCA) is thought to be highest among elite athletes because of greater exposure to environmental factors (including strenuous exercise and dehydration) compared with the general population. Athletes can also experience career-, life- or limb-threatening injuries. Such events fall under the umbrella of “critical” incidents because of both the risk to life and “their potential to create significant human distress that can overwhelm an individual’s usual means of coping” (9).

Instances such as sudden collapse of Christian Eriksen during the 2021 European Football Championships and the fatal neck injury sustained by Adam Johnson from a skate during an ice hockey collision in 2023 highlight the importance of having plans to manage such critical incidents. Accordingly, clinicians working in sport should have robust measures in place to mitigate risk and maximise the likelihood of successful outcomes.

This publication serves as a practical guide to suggest how sporting organisations can

(i) plan effectively for critical incidents;
(ii) manage them effectively; and
(iii) provide follow-up care for all those involved in critical incidents.

This publication is supported by the Faculty of Sport and Exercise Medicine UK (FSEM), reflecting its alignment with current best practice in sport and exercise medicine.

## Pre-event planning and emergency action plan

While the absolute risk of critical incidents is low (10), their impact and implications are often significant for the individuals involved, their families and friends, and the sporting organisations. In addition to ensuring that staff possess relevant accreditation (including qualifications in pre-hospital emergency care and trauma management and evidence of reflective practice) and medical teams are provided with opportunities to practice potential scenarios as a team, clinicians and sporting bodies should ensure that emergency action plans (EAPs) are available for each environment in which athletes train and compete. While there is no universally agreed standard for the content of EAPs, Table 1 outlines key components informed by a recent scoping review conducted by the authors, alongside existing literature and expert consensus in this area.^1^

**Table 1:** Core components/details of an EAP.

Component	Key details to capture
Location	Address, what3words/GPS coordinates, access routes, local hospitals (including specialist centres for cardiac pathology, major trauma)
Breadth of potential injuries	Protocol for spinal extrication, major MSK pathology, and SCA
Coordination of emergency medical staff and local medics	Meeting points, location of medical rooms, and exit routes
List of equipment	Location and how to summon (hand signals, etc.)
Outline of roles	Situation-specific (e.g., spinal trauma, SCA) and expectations relating to how often scenarios should be practiced
Protocol for post-incident	Review procedures and support structures
Miscellaneous	Contact details for key personnel (lead doctor, lead paramedic, local hospital)

MSK, Musculoskeletal.

EAPs should not only be accessible and visible to those working in sport, but best practice would also include regular rehearsal and sharing with all staff members, including any non-clinical staff who may be expected to have a role in managing the scene surrounding a critical incident. Consideration should also be given to sharing these and inviting input from a wide range of stakeholders (including athletes, coaches, executives, and board members), as responsibility lies across the whole organisation and important insights might be gleaned by involving professionals with broad skill sets and considerations.

EAPs should also take into account any unique environmental challenges provided by their sport or competition arena, examples of which are highlighted in Box 1. From a practical perspective, copies of EAPs should also be provided to any visiting medical teams attending fixtures/events to allow familiarisation with the details in advance. For those regularly working in one location, consideration should be given to building relationships with local hospitals to help streamline care.

Box 1 **Event types or sports with unique challenges.**

**Table:** 

**Water sports**: equipment and water-associated risks; potential remoteness of medical provision
**Equestrian**: horse-related risks and risk of secondary impact(s)
**Road cycling:** road “furniture” and courses that traverse borders and different geo-political regions; potential remoteness of medical provision.
**Motorsport:** high-velocity trauma

## Post-traumatic care protocols

Whilst EAPs can maximise the likelihood of a positive outcome and help manage the scene at the time of the incident, sporting bodies and clinicians should also take into account the potential detrimental consequences associated with observing or being involved in a critical incident.

This current author group recently published a call to action to ensure that sporting bodies routinely discuss and document protocols for managing the post-traumatic period in elite sport. Five principles, termed “the 5Cs,” were highlighted, which could be implemented following a critical incident in sport (11):

- Communication: Clear, timely, and structured communication should be established with all stakeholders, including athletes, staff, and next of kin, with defined roles and responsibilities to ensure accuracy and consistency of information.
- Care: Immediate and ongoing support should be provided to all affected individuals, including athletes, staff, and clinicians, with attention to both physical and psychological wellbeing.
- Calm: A composed and structured environment should be maintained following the incident through clear leadership, role clarity, and adherence to established protocols.
- Connectedness: Individuals involved should be supported in maintaining connections with teammates, staff, and support networks to reduce isolation and promote collective coping.
- Contemplate return to play: Decisions regarding return to training and competition should be individualised, clinically guided, and consider both physical recovery and psychological readiness.

Once the immediate incident has been managed, a “hot debrief” of the event is often helpful for all those directly involved, and the team should be encouraged to stay together to support each other. The next of kin (NoK) should be contacted urgently, with a staff member assigned to inform them of events. This communication should take place within hours of the incident.

Beyond this acute phase, individuals may begin to exhibit varying emotional responses. Sporting bodies should consider a secondary “cold” debrief, involving the medical team within weeks of the event, to help identify key learnings from the incident (both technical and non-technical) and address any queries that may have emerged. Such a debrief may be best guided by a trained mental health professional. These debriefs can also be integrated with any “significant event analysis” forms and processes that may exist as part of the wider governance framework, while learnings may be shared across the organisation to highlight reflective practice and support the continuous improvement of care standards.

In the medium to long term, those involved in the incident should continue to receive support, and those considered at risk of developing mental health conditions, including depression, anxiety and post-traumatic stress disorders (PTSD), should be monitored, with support provided by formal mental health professionals as required. Further support may also be provided to the wider team, as returning to play may cause people to re-experience the event.

## Summary

Preparing for critical incidents should be seen as best practice for sporting bodies or clinicians working in elite sport. Detailed planning to mitigate risks (including athlete screening), together with practical, robust, and rehearsed EAPs, should also be considered key deliverables and part of “gold-standard” practice.

Protocols are well established in sports medicine and provide a framework for managing difficult situations in a standardised fashion. Post-incident protocols could be proactively developed and discussed with all relevant stakeholders, in the same way that EAPs have been embraced by a range of stakeholders. Such a step will likely minimise the risk of harm from critical incidents and maximise the likelihood of recovery and positive outcomes for those involved.

## Footnotes

^1^Morgan K, Heron N. A scoping review of protocols for managing catastrophic injuries to athletes during sporting events [in review].
