# Correction: Standardising the preparation for, and delivery of care during, and after critical incidents: a Faculty of Sport and Exercise Medicine UK publication

**DOI:** 10.3389/fspor.2026.1903538

**Published:** 2026-06-24

**Authors:** Neil Heron, Steffan Griffin

**Affiliations:** 1 Centre for Public Health, Queen’s University Belfast, Belfast, United Kingdom; 2 SailGP, London, United Kingdom; 3 Physical Activity for Health Research Centre, University of Edinburgh, Edinburgh, United Kingdom

**Keywords:** athlete safety and welfare, critical incidents, emergency action planning, post-traumatic care, sports medicine

The title of this article was erroneously given as: Standardising the preparation for, and delivery of care during, and after critical incidents: a Faculty of Sport and Exercise Medicine position statement. The correct title of the article is: Standardising the preparation for, and delivery of care during, and after critical incidents: a Faculty of Sport and Exercise Medicine UK publication

A correction has been made to the **Abstract**:

“Participation in sport provides substantial physical and mental health benefits; however, it also carries a risk of rare but high-impact critical incidents. This policy brief outlines a practical, standardised framework for the preparation, management, and follow-up of such incidents in sport. Drawing on current evidence and expert consensus, we emphasise the importance of robust, rehearsed emergency action plans tailored to specific environments, alongside structured post-incident care incorporating communication, debriefing, and psychological support through the “5Cs” approach. This publication is supported by the Faculty of Sport and Exercise Medicine UK (FSEM), reflecting its alignment with current best practice in sport and exercise medicine. By integrating prevention, acute response, and rehabilitation, this work provides actionable guidance to improve athlete safety and promote consistent, high-quality care across diverse sporting contexts.”

A correction has been made to the section **Introduction**, Paragraph 4:

“This publication serves as a practical guide to suggest how sporting organisations can

- plan effectively for critical incidents;
- manage them effectively; and
- provide follow-up care for all those involved in critical incidents.

This publication is supported by the Faculty of Sport and Exercise Medicine UK (FSEM), reflecting its alignment with current best practice in sport and exercise medicine.”

The original version of this article has been updated.

